# Thermal Effects in the Ablation of Bovine Cortical Bone with Pulsed Laser Sources

**DOI:** 10.3390/ma12182916

**Published:** 2019-09-09

**Authors:** David Canteli, Cristina Muñoz-García, Miguel Morales, Andrés Márquez, Sara Lauzurica, Juan Arregui, Aritz Lazkoz, Carlos Molpeceres

**Affiliations:** 1 Centro Láser, Universidad Politécnica de Madrid, Alan Turing 1, 28031 Madrid, Spain; 2 Deneb Medical, Paseo Mikeletegui, 83, 20009 San Sebastián, Spain

**Keywords:** bone osteotomy, laser ablation, ablation rate, hard tissue, ns-laser, ps-laser, thermal effects

## Abstract

Lasers have advantages as bone surgical tools over mechanical methods, but two goals should be achieved to assure its use: Similar ablation rates to those obtained with mechanical tools (1 mm^3^/s at least) and to avoid thermal damage, a condition that can prevent proper bone healing. We present results of cow femoral bone with a 355 nm nanosecond (ns) and a 1064 nm picosecond (ps) pulsed laser sources that allow us to discuss the influence on the process of pulse duration and the selective ablation through high energy absorption (as bone highly absorbs 355 nm radiation). The treated samples were characterized by scanning electron microscopy (SEM) and Raman spectroscopy. The evaluation of the thermal effects produced in the samples shows clear differences between both laser sources: On one hand, the ns laser allows reaching high ablation rates (around 1 mm^3^/s); Raman spectra show no signal of bone carbonization, but unavoidable thermal effects in the form of melted and solidified material have been observed by electron microscopy in the samples treated with this laser. On the other hand, ablation without any sign of thermal effects is obtained using the ps laser, but with lower ablation rates, (around 0.15 mm^3^/s).

## 1. Introduction

Instruments to cut bone have changed throughout history, from saws and drills to the modern piezoelectric osteotomes, but the procedure has been always the same: to generate mechanical stress onto the bone surface until the tool penetrates it. Leaving aside the obvious advantages in precision and control of the modern instruments, all of them present disadvantages, such as mechanical damage induced in the bone, possible breaking of the head of the instrument, or it being stuck to the bone.

Lasers have advantages as surgical tools, such as non-contact processing, a very precise ablation of tissues, with incisions around 100 µm wide, and with the possibility of using computer-controlled systems. Although, in recent years, there has been an intense effort in the study and development of laser tools, such as surgical knives and scalpels, and while having multiple promising applications in different fields like osteotomy, craniotomy, spine surgery, or craniomaxillofacial surgery [1], the use of mechanical saws, milling cutters, and drills is still preferred over lasers. There are two reasons for this preference: First, potential thermal effects introduced in the tissues by the laser process should be avoided; thermal damage and carbonization can prevent the proper bone healing [2,3,4], with other associated problems as thermomechanical cracking [5]. As the bone healing is a critical aspect for any application in surgery, avoiding thermal damage becomes the main goal for this kind of processes. A usual way to diminish any possible thermal effect is the use of hydration (by water spray irrigation [6,7] or even by submersion of the sample [8]). Second, it is necessary to achieve an acceptable and comparable material ablation rate. Mechanic tools allow an ablation rate over 1 mm^3^/s, so to be in a competitive position, a similar ablation rate value with lasers is desirable. The importance of this can be seen in the literature, with plenty of publications about the ablation rate of bone with a wide variety of laser systems, with wavelengths ranging from ultraviolet to mid infrared, and any kind of pulse duration: Continuous wave, microseconds [5,6,7], nanoseconds (ns) [9], picoseconds (ps) [8,10], and femtoseconds [11,12,13].

The best results in the removal or ablation of hard tissues by laser have been obtained with two types of lasers that produce a very different ablation process. On one hand, pulsed lasers with a pulse duration of several hundreds of microseconds emitting in the short infrared wavelength, around 2.8 µm, where the strong absorption of water leads to its evaporation and the mechanical break of the tissue due to the generated shockwave. Irradiation of hard tissues with these lasers shows high ablation rates and low thermal damage, but present some problems like sub-superficial cracking, or tissue necrosis at some degree [14,15]. On the other hand, ultrafast laser sources, although with ablation rates lower than the obtained with mechanical tools, produce an ablation with almost none of the thermal effects [10,11,16,17].

In this paper, we study the role of material absorption and pulse width in the ablation of femoral bone cow. Bone is mainly composed of collagen and hydroxyapatite [18]. Both components show higher absorption at 355 nm than at 1064 nm wavelengths, with collagen having an absorption maximum near 355 nm [19,20,21,22]. To do the study, we selected two laser sources: One emitting at 355 nm, corresponding to the absorption peak of collagen, and with a pulse duration in the ns regime associated with a more thermic process; and the other emitting at 1064 nm, where the absorption coefficient is lower but pulsed in the ps regime. This allow us to discuss the effect of the selective absorption of the material depending on the wavelength with the effect of the pulse duration, as well as their role in the thermal effects introduced in the bone during the laser process.

Having in mind the two main parameters stated for a successful application (thermal effects and ablation rate), we present a detailed morphologic characterization of the treated samples and calculate the ablation rates obtained with each laser. We also discuss the different morphology changes generated by the thermal effects that appear with each laser system.

## 2. Materials and Methods

For the ablation experiments, fresh bovine femur was cut using a band saw into squared pieces of approximately 5 cm × 5 cm to improve sample handling. There is no standard way to store the bone samples, with different options ranging from storing in Elomel solution [11], distilled water [12], kept moist with physiological saline [10], or with no special storing [6,23]. This is not a critical issue as no differences in the ablation experiments have been found between samples stored in formalin, samples maintained at −18 °C and defrosted, and samples kept at room temperature [8]. As for the samples preparation, some works also show a polishing of the samples previous the laser ablation [12], and others left the bones without polish [10,11]. We decided to maintain the samples in a watertight container at 4 °C prior the laser tests and to maintain the samples with as similar characteristics as possible to a real sample; previous to the laser ablation, the periosteum and soft tissues were removed with a scalpel, but no additional polishing or cleaning treatment was carried out apart from water washing.

Two different laser systems have been used in this study: An ns laser source working at a wavelength of 355 nm and with a pulse duration about 20 ns (PULSEO, a Nd: YVO_4_ solid-state laser from Spectra Physics, Santa Clara, CA, USA); and a ps laser source with a wavelength of 1064 nm and a pulse duration about 12 ps (Atlantic, a Nd:YAG from Ekspla, Vilnius, Lithuania). As one of the objectives of the experiment was to have a high ablation rate, we used a scan head to deliver the laser beam to the sample, allowing scanning speeds up to several meters per second. An f-theta lens with focal length of 250 mm was used for the ns laser, and another with focal length of 254 mm was used in the ps system, leading to a 1/e^2^ beam radius in sample of 15 µm and 19 µm, respectively. A crisscross pattern, obtained by scribing parallel grooves with a constant separation between them and a second array of parallel laser scribes perpendicular to the former ones, was used to ablate squared areas of 2 mm × 2 mm. This pattern allows the change of deposited energy in an easy way by just varying the pulse energy, scanning speed, or pattern pitch, and leads to a quite homogeneous distribution of the energy deposited in the material, regardless the used parameters. The areas obtained with both laser systems using this crisscross pattern show a homogeneous aspect and depth, and we found no difference between the points were different laser process crosses with the others. There are several parameters that affect the process: Laser pulse frequency, laser beam speed, laser pulse fluence, process pitch (distance between parallel laser processes), and number of cycles (the number of times the sample area is treated with the laser). Table 1 shows the range of parameters used. We include values of overlap defined as *O_p_* = 100(1 − *d*/(2*ω*_0_)), being *ω*_0_ the 1/e^2^ beam radius, and *d* the distance between adjacent laser pulses. High overlap values favor the carbonization of the sample more in the ns regime than in the ps, allowing the ps system the use of higher overlap values.

As the easier approximation to the removal rate problem, we decided to fix the focal plane of the laser beam at the sample surface, without adjusting it during the laser process. This means that as the number of cycles is increased the efficiency of the process drops. The use of an adequate mechanism of focal plane adjustment will lead to higher depths and removal rates. The values of depth and removal rate shown in this work can be considered as a minimum with the used process parameters.

The morphology of the samples was characterized by optical confocal microscopy (Leica DCM 3D, Leica microsystems, Wetzlar, Germany). This system provides a 3D topography of the sample, allowing the measurement of the depth of the removed areas and the calculation of the ablated volume, as well as the ablation rate (the relation between ablated volume and processing time). Also, images of the samples were taken using a HITACHI (Tokyo, Japan) S-3500N scanning electron microscope (SEM). To avoid the accumulation of static charges, the samples were coated with a gold-palladium film deposited by DC sputtering. The SEM images included in this paper have been taken at the bottom of the treated areas. Finally, Micro-Raman spectroscopy (InVia Renishaw, Wotton-under-Edge, UK), using the 514.4 nm line of an Ar laser) was used to study the composition of the treated and non-treated samples. The spectra were obtained working at 10% of laser power with an acquisition time of 30 s and 20 accumulations. As a clarification, we are going to use two different terms when dealing with the damage induced on the samples. Carbonization means some degree of darkening of the surface, while thermal damage is used when there is no darkening and the damage is seen only under the microscope.

## 3. Results

### 3.1. Ablation Rate

**Figure 1:**
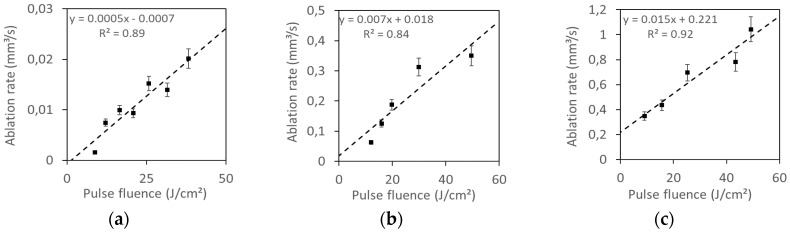
Ablation rate as a function of pulse fluence: Taken from the experiments with the ultraviolet (UV) nanosecond (ns) laser pulses for three different pulse repetition rates: (**a**) 2 kHz; (**b**) 30 kHz; (**c**) 60 kHz.

**Figure 2:**
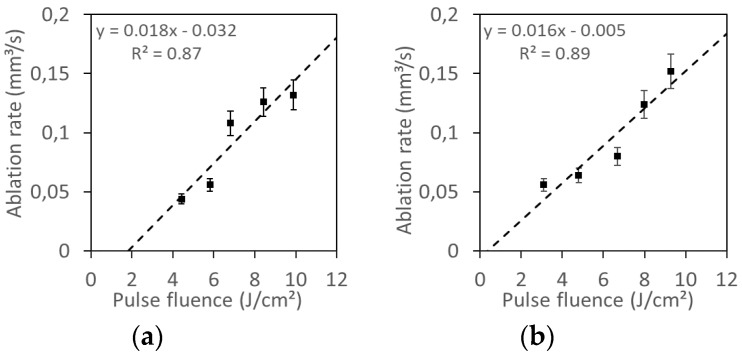
Ablation rate as a function of the pulse fluence from the experiments with the infrared (IR) picosecond (ps) laser pulses for two different pulse repetition rates: (**a**) 100 kHz; (**b**) 600 kHz.

The main objective of these experiments was to obtain an ablation rate as high as possible without thermal damage in the samples. Figure 1 and Figure 2 show the ablation rate, calculated as the total ablated volume divided by the processing time, obtained for different maximum pulse fluences at different pulse repetition rates (with fixed process speed, pitch, and number of cycles). As it can be seen, the ablation rate rises as the pulse fluence is increased, and a linear dependence is found in all cases. Similar behavior has been found when working with other laser sources [10,11].

As it can be seen from Figure 1 and Figure 2, the ps laser shows very little difference between the results obtained with 100 kHz and 600 kHz, meanwhile for the ns laser the ablation rate strongly depends on the used frequency.

### 3.2. Morphology Characterization and Thermal Damage

**Figure 3:**
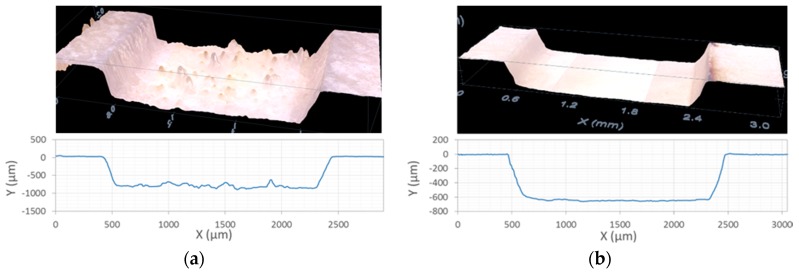
Confocal microscope images and profiles of 2 mm × 2mm squared holes ablated with: (**a**) UV ns laser pulse, with 880 µm deep; (**b**) IR ps laser pulse, with 690 µm deep. The profiles correspond to a line crossing the center of the square. The pulse fluence, process speed, repetition frequency, pitch, and number of cycles used for each sample are, respectively: (**a**) 16.6 J/cm^2^, 50 mm/s, 2 kHz, 50 µm, 50; (**b**) 11.9 J/cm^2^, 500 mm/s, 100 kHz, 10 µm, 20.

**Figure 4:**
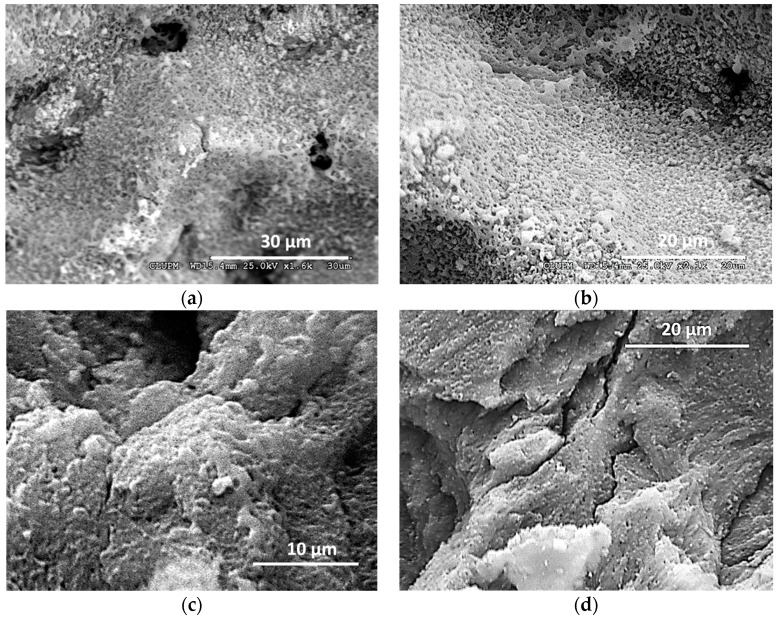
SEM images of bone samples ablated using the UV ns laser pulse. Melted material (rounded shapes) is an indication of thermal damage. The pulse fluence, process speed, repetition frequency, pitch, and number of cycles used for each sample are, respectively: (**a**) 38.2 J/cm^2^, 6000 mm/s, 100 kHz, 50 µm, 100; (**b**) 49.2 J/cm^2^, 4000 mm/s, 60 kHz, 50 µm, 80; (**c**) 49.2 J/cm^2^, 4000 mm/s, 60 kHz, 50 µm, 20; (**d**) 44.6 J/cm^2^, 2000 mm/s, 20 kHz, 50 µm, 50.

**Figure 5:**
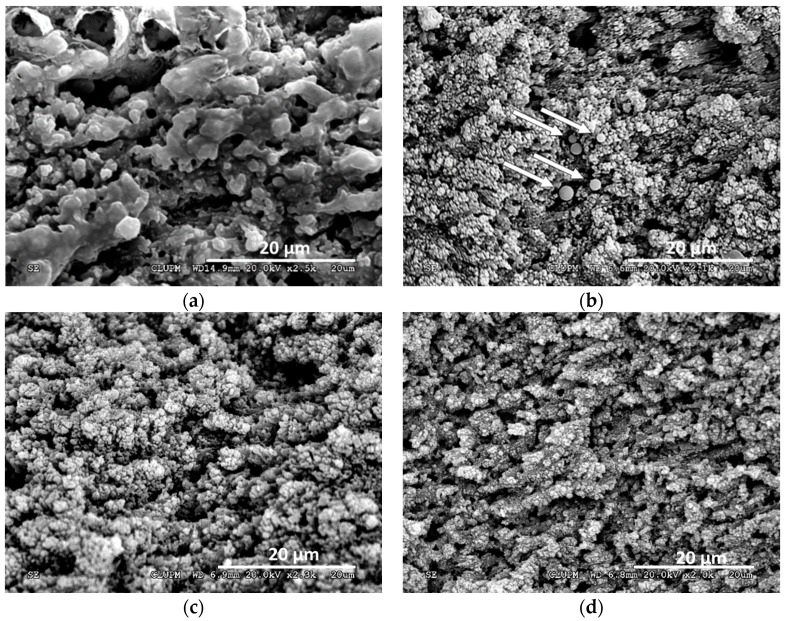
Different SEM images of bone samples ablated using the IR ps laser source. Thermal damage in the form of rounded shapes is clearly visible in images (**a**) and (**b**). The pulse fluence, process speed, repetition frequency, pitch, and number of cycles used for each sample are, respectively: (**a**) 13.9 J/cm^2^, 5000 mm/s, 400 kHz, 20 µm, 20; (**b**) 13.9 J/cm^2^, 500 mm/s, 100 kHz, 20 µm, 20; (**c**) 9.9 J/cm^2^, 500 mm/s, 100 kHz, 20 µm, 20; (**d**) 8.4 J/cm^2^, 500 mm/s, 100 kHz, 20 µm, 20.

Although, enough laser fluence both lasers produce the carbonization of the sample surface, some differences were observed: With the ns laser, a gradual brown coloration is produced as the pulse fluence is increased, before the complete carbonization of the sample. This is not observed when using the ps laser, where total carbonization of the area appears in an abrupt way. To have a better idea of the presence and extent of the thermal damage, the morphology of the samples was characterized using confocal microscopy and SEM (Figure 3, Figure 4 and Figure 5).

#### 3.2.1. UV NS Laser

When using the UV ns laser (see Figure 3a), the holes present a homogeneous depth, showing a rough bottom with debris and in some samples, redeposited material in the hole edges. The apparition of carbonization heavily depends on the energy deposition rate: Low repetition rates (2 kHz, 40 mm/s) lead to material ablation without darkening of the surface, regardless of the pulse energy and cycles used, but with very low ablation rates. To obtain higher ablation rates without carbonization of the surface higher pulse repetition rates and higher process speeds are needed (6000 mm/s for 100 kHz). With an adequate selection of parameters a high ablation rate is reached with no darkening of the surface, but all samples ablated using the UV ns laser present in its surface signs of melted and redeposited material. Figure 4 shows SEM images of different samples were clear signals of melted material could be observed.

#### 3.2.2. IR PS Laser

The ablation with the IR ps laser lead to holes with a smoother and more homogeneous bottom (see Figure 3b). Under the SEM microscope, some of the samples with no visible thermal damage show thermal effects as partially melted structures (image Figure 5a) or the apparition of small spheres of melted material (image Figure 5b). Adequate process parameters lead to holes without any trace of thermal damage. Figure 5c,d are examples of those undamaged samples were the bottom of the craters do not show any melted material nor ablation debris and the bone structure is preserved.

**Figure 6:**
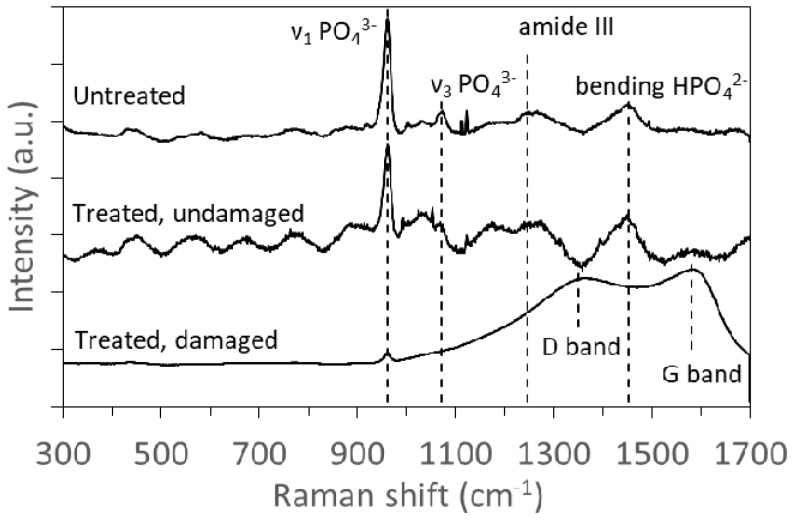
Normalized Micro-Raman spectra of untreated bone, and laser-ablated samples without visible thermal damage (treated, undamaged) and with visible thermal damage (threated, damaged).

As an additional method to analyze the damage in the samples and detect the thermal damage, Micro-Raman spectroscopy was used. Figure 6 shows an example of the obtained Micro-Raman spectra in three different samples: A non-treated sample, a treated sample were no thermal damage appears, and a sample with signs of thermal damage. The spectra related to the untreated sample and the treated sample without thermal damage are very similar, showing an intense band at 960 cm^−1^ (ν_1_ vibration band of PO_4_^3−^), and other less intense at 1070 cm^−1^ (ν_3_ vibration band of PO_4_^3−^), 1247 cm^−1^ (amide III collagen band), and 1453 cm^−1^ (bending vibration band of HPO_4_^2^^−^), all of them related to bone [16,24]. The sample with thermal damage shows two broad bands around 1350 cm^−1^ and 1580 cm^−1^, corresponding to the graphite bands ‘D’ and ‘G’, and related to amorphous carbon [16,25], showing there has been some carbonization of the bone material.

## 4. Discussion

**Figure 7:**
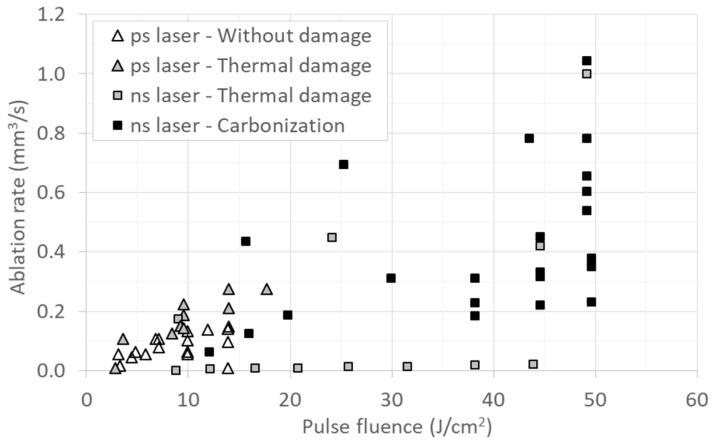
Ablation rate versus laser pulse fluence for samples obtained with the UV ns and IR ps lasers, classified by the thermal damage observed.

Considering all these results it is possible to represent the treated samples depending on the thermal effects observed. Figure 7 represents ablation rate versus laser pulse fluence for the samples processed with both laser sources, and classified by the thermal effects observed: The samples obtained with the UV ns laser divided in those with thermal damage but no carbonization signs (grey squares), and those with carbonization signs (black squares). The IR ps samples divided by those with thermal damage (grey triangles) and the ones without damage (hollow triangles). As said before, the laser fluence is not the only process parameter, and different ablation rates and thermal effects can be obtained depending on the processing speed, laser frequency and pitch, but this representation allows the discussion of the obtained results.

Table 2 includes the process parameters and ablation rates of the samples showing better results for the UV ns (samples with thermal damage but no signs of carbonization) and IR ps lasers (samples without thermal damage).

Most of the samples obtained with the UV ns laser show some carbonization, and all samples present some thermal damage in the form of melted material when observed by the microscope, while the use of the IR ps laser source allows to avoid any thermal damage. This (as well as the influence of pulse repetition frequency observed in Figure 1 and Figure 2, where the ablation rate strongly depends on the used frequency for the ns laser but there is no influence for the ps laser) will be related with the different interaction between light and matter for the ns pulses and the ps pulses.

The energy is absorbed by the electrons and transferred to the sample lattice by a carrier-phonon scattering process on a ps timescale. For ns laser pulses the pulse duration is several orders of magnitude higher, and the laser can be considered a heat source, while in the case of ultrashort laser pulses, with much higher intensities, other processes as multiphoton absorption and avalanche ionization become relevant. These processes increase the number of free electrons, changing dramatically the optical properties of the material. The initial absorption coefficient of the material play a minor role under ultrashort laser pulses compared to long pulse duration lasers [13,22]. Although the ps laser pulses are not short enough to avoid any thermal effect, the heating of the sample is drastically reduced [26].

Regarding the ablation rate, with the ns laser it is possible to obtain, with adequate parameters, ablation rates near 1 mm^3^/s with no carbonization signals. Although some thermal damage is always present in the form of melted material, these ablation rates are high enough to compete with the mechanical tools in bone ablation. On the other hand, the ablation rates obtained with the ps laser are around 0.15 mm^3^/s, similar to those observed in the literature with ps or other ultrafast laser sources [8,10,11,25,27] but low compared with the mechanical tools’ throughput.

As thermal damage produced during the laser process could affect the bone healing, the promising ablation rates reached with the ns laser should be taken with caution. A practical solution, previously suggested in literature [28], will be to adjust the laser process to optimize the results, in this case using both systems to first remove most of the tissue with the ns laser at a high removal rate and with a limited thermal damage, and then eliminate that damaged area with the ps laser, thus resulting in an ablation process with minimal thermal damage and maximal ablation speed. Nevertheless, any laser process should be checked by in vivo tests to study its impact on bone healing.

## Figures and Tables

**Table 1:** Range of the different process parameters used. ns = nanosecond; ps = picosecond.

Laser Source	Frequency kHz	Speed mm/s	Fluence J/cm^2^	Pitch µm	Cycles	*O_p_* %
PULSEO (355 nm, ns)	2–100	50–7000	8.8–49.6	50–100	20–100	0–20
Atlantic (1064 nm, ps)	100–600	100–5000	2.8–17.7	10–20	20–60	0–98

**Table 2:** Process parameters leading to better results with both lasers, and ablation rates.

Laser source	Frequency kHz	Speed mm/s	Fluence J/cm^2^	Pitch µm	Cycles	Ablation Rate mm^3^/s
PULSEO (355 nm, ns)	60	4000	49.2	50	20	1.00
	30	3000	24.1	50	50	0.45
	20	2000	44.6	50	50	0.42
	60	4000	9.0	50	40	0.17
Atlantic (1064 nm, ps)	100	500	13.9	10	20	0.15
	100	500	11.9	10	20	0.14
	100	500	9.9	10	20	0.13
	400	5000	7.1	10	20	0.11
